# Valproate Exposure *in ovo* Attenuates the Acquisition of Social Preferences of Young Post-hatch Domestic Chicks

**DOI:** 10.3389/fphys.2019.00881

**Published:** 2019-07-16

**Authors:** Gergely Zachar, András S. Tóth, László I. Gerecsei, Sándor Zsebők, Ágota Ádám, András Csillag

**Affiliations:** ^1^Department of Anatomy, Histology and Embryology, Faculty of Medicine, Semmelweis University, Budapest, Hungary; ^2^Department of Systematic Zoology and Ecology, Institute of Biology, Faculty of Science, Eötvös Loránd University, Budapest, Hungary

**Keywords:** autism spectrum disorder, avian, social cohesion, embryonic development, developmental disorder, social brain network

## Abstract

Embryonic exposure to valproic acid (VPA) is known to produce sociability deficits, resembling human autistic phenotypes, in several vertebrate species. Animals living in groups prefer the proximity of peers and have the ability to perceive and to respond to social signals for modifying behavior. Chicks of Galliform birds, known to display early preference behaviors, have been used extensively for adaptive learning studies. Young precocial birds seem to be useful models also for studying the effect of embryonic VPA treatment. Here, domestic chicken eggs were injected with sodium valproate (200 μl of 35 μmol/L solution) or with vehicle (distilled water) on the 14th day of incubation. After hatching, the chicks were tested for one-trial passive avoidance learning at day 1, vocalization due to isolation as a measure of stress level (day 2), approach preference to large versus small groups of age-matched conspecifics (day 5), and to those with normal versus blurred head features (day 7). In addition, we tested the preference of birds to conspecifics reared in group versus those reared in isolation (day 9), as well as the preference of chicks to familiar versus non-familiar conspecifics (day 21). Our findings confirm previous reports concerning an adverse effect of VPA on embryonic development, including a tendency for aborted or delayed hatching and, occasionally, for locomotor disorders in a small percentage of birds (eliminated from later studies). Otherwise, VPA treatment did not impair motor activity or distress level. Memory formation for the aversive stimulus and discrimination of colors were not impaired by VPA treatment either. Innate social predispositions manifested in approach preferences for the larger target group or for the birds with natural facial features remained unaffected by VPA exposure. The most prominent finding was attenuation of social exploration in VPA-exposed birds (expressed as the frequency of positional switches between two stimulus chicks after the first choice), followed by a deficit in the recognition of familiar conspecifics, unfolding at the end of the third week. Social exploration and recognition of familiar individuals are the key elements impaired at this stage. The results underline the importance of early social exploration in ASD.

## Introduction

Autism spectrum disorder (ASD) is currently envisaged as a neurodevelopmental disorder associated with altered social behavior. In addition to a multitude of human studies, it was necessary to find reliable animal models for investigation into various aspects of ASD. One such model for laboratory rodents is prenatal exposure to valproic acid (VPA), a known antiepileptic substance and mood stabilizing agent (Rodier et al., 1996; Schneider and Przewlocki, 2005; Wagner et al., 2006; for recent reviews, see Roullet et al., 2013; Nicolini and Fahnestock, 2018). Social impairment accompanies all forms of ASD. *In utero* VPA causes sociability deficits postnatally in the adult animals (Roullet et al., 2010; Kim et al., 2011; Moldrich et al., 2013), and VPA-treated animals are also known to exhibit anxiety, depression-like behavior, and abnormal nociception thresholds (Wu et al., 2017). Embryonic exposure to VPA was found to produce an autistic-like phenotype even in fish (Baronio et al., 2017; Chen et al., 2018). The mechanism of action of VPA is rather complex but the key factor eliciting developmental and behavioral alterations seems to be that VPA is an inhibitor of histone deacetylase (Moldrich et al., 2013), potentially affecting gene expression and transcription engaging the Wnt1 signaling pathway, a robust “caudalizing” factor in rostrocaudal patterning of the developing CNS (Wiltse, 2005; Jang and Jeong, 2018).

Social behavior has been a widely studied phenomenon in life sciences. Most of the studies investigating the neurobiological bases of sociability exploit the fact that animals need to form bonds, at least temporarily, in order to reproduce. These bonds may represent strong contacts between sexual partners during or even beyond the time of mating, or between parents and offspring. The basis for such bonding may be hardwired innate preferences and affections and/or specific early learning mechanisms such as filial (Lorenz, 1937; Cherkin, 1969; Di Giorgio et al., 2016) and sexual imprinting (Irwin and Price, 1999). It is well known that many animals living in groups are often linked stronger to group members than to other conspecifics, they can even form stronger coalitions (often based on genetic relatedness) within such groups (Henzi and Barrett, 1999; Bergman, 2010). Effective cooperation within a group requires the preference for proximity of group members, suppression of aggression toward conspecifics, ability to perceive and respond to social signals and to change (often synchronize) behavior accordingly (Anacker and Beery, 2013). Group cohesion and evaluation of socially relevant information have been implicated as essential factors in various animal models of autism (Seebacher and Krause, 2017).

Earlier studies on pair bonding and, less extensively, on parent-offspring relations, have identified a network of brain regions associated with social behavior (Goodson, 2005). Present in a wide variety of vertebrate animals, this network seems to regulate most of the social interactions in these relations (O’Connell and Hofmann, 2011). Less investigated, the neural correlates of group forming (Goodson et al., 2009; Goodson and Kingsbury, 2011), group cohesion, and responsiveness to group signals in more general terms (not only in those of reproductive significance) might well engage the same brain regions and follow the same organizational principles (Wilson et al., 2016). In the present study, we focus our attention on the latter category.

The choice of the domestic chick as a model species for the current investigation was based on the assumption that certain key phenomenological elements of sociability, essential for survival, are associated with distinct neural systems that are common to vertebrate animals. Based upon previous findings in mammals, we chose to investigate another, phylogenetically distant species, the domestic chicken. Newly hatched chicks of Galliform birds are known to display various types of preference behavior, e.g., genetically determined preference for color of Japanese quail (Kovach, 1980; Csillag et al., 1995), or predispositions toward social stimuli of domestic chicks (Zachar et al., 2008, 2016; Di Giorgio et al., 2016). A precocial nidifugous species with a remarkably mature brain structure at hatching, the domestic chicken has long been used for investigation of early adaptive learning (imprinting or passive avoidance training, for an overview, see Rose, 2000), and it also appears to be a useful model for studying the effect of embryonic VPA treatment on acquired and innate behaviors. Traditionally, domestic chicken eggs have long been used for developmental studies and *in ovo* manipulations, and this propensity could be exploited here by injecting the eggs with VPA or vehicle at the critical time of development.

VPA has been examined in birds as a teratogenic agent (Whitsel et al., 2002; Tureci et al., 2011; Hsieh et al., 2013; Akhtar et al., 2015). Concerning ASD, administration of VPA into the egg caused an impairment of social behavior (but not of imprinting) in chicks (Nishigori et al., 2013). In a recent study (Sgadò et al., 2018) on visually naïve young post-hatch chicks (1–3 days of age), VPA treatment *in ovo* was found to attenuate the manifestation of the innate predisposition for an inanimate chicken (stuffed hen) over an object with scrambled features of chicken. This effect on (socially related) visual predisposition was not accompanied by a similar impairment of filial imprinting, in agreement with the Nishigori study (Nishigori et al., 2013). Recently, Lorenzi et al. (2019) showed that innate preference for an object autonomously changing speed over one moving with a constant speed was affected in chicks by embryonic VPA treatment. While it is increasingly evident that domestic chicks might offer a useful and inexpensive alternative model for studies of autism-related social deficits, the aptitude of the model could be further corroborated by a refined analysis of the affected elements behaviors, also extended to a later period of postembryonic maturation. In the previous studies little attempt has been made to test whether the impairment of the social behavior is a part of a more general attenuation of locomotor activity or cognitive deficit. Also, it was described that VPA negatively influences the group-forming and group-cohesion of young chicks (Nishigori et al., 2013), however, it is unknown whether the recognition of the siblings as social stimuli was attenuated, or else social exploration and social learning as such were impaired. In the present study, we tested the role of innate preferences, as well as the learned component of social recognition in chicks treated *in ovo* with VPA from hatching to 3 weeks of age. Moreover, we intended to exclude the role of aspecific effects of VPA by using behavioral tests for early learning and non-social stimulation, such as the presence of a predator, to corroborate the validity of the VPA model in chicks. By so doing, we are focusing primarily on the early development of social recognition and the dichotomy between innate and acquired preferences.

## Materials and Methods

### Animals

Eggs of Hunnia Broiler hybrid chicks were purchased from a local distributor (Bábolna Bio kft.) and placed into an incubator kept at 37.8°C with 55% relative humidity, reduced to 37.5°C after the first week and, finally, to 37.3°C 3 days before hatching (during this last period the relative humidity was 70%). The hatching temperature was continuously monitored by a cell phone based remote surveillance application. Eggs containing live embryos were selected by candling on days 7 and 13, discarding the eggs that showed no signs of development. On the 14th day of the incubation, 200 μl of 35 μmol/L sodium valproate (dissolved in sterile, pyrogen-free distilled water) was injected into the air sac of 32 eggs. The rest of the eggs (42) were injected with 200 μl of the above vehicle only, and served as controls. Injections were performed similarly to the methods described in previous studies (Nishigori et al., 2013; Sgadò et al., 2018). Briefly, the eggs were placed on a supporting cup, with the air sac (previously located and marked) facing upwards. The egg shell was wiped with a disinfectant solution, allowed to dry and perforated with a sterile hypodermic needle. Then, the solutions were injected manually, using a small gauge hypodermic needle and syringe, slowly enough to avoid backflow and steadily enough not to penetrate the inner shell membrane. After retraction of the hypodermic, the burr hole was sealed with candle wax. All animals were kept and treated according to the regulations of the ethical committee of the Semmelweis University, and all experiments were approved by the Ethical Committee on Animal Experimentation, and permitted by the Food Chain Safety and Animal Health Directorate of the Government Office for Pest County (Permit Number: XIV-I-001/2269-4/2012). Procedures were in harmony with the EU Council directives on laboratory animals (86/609/EEC).

### Behavioral Tests

We performed six different behavioral tests on 26 of the control and 18 of the VPA treated chicks. The rest of the controls were used as stimulus chicks in the choice between isolation-reared and group-reared individuals. Of these, nine were kept isolated and 6 as a group. Ambient temperature in the experimental room was 30°C.

#### Taste Aversion Learning and Positive Reinforcement (Day 1)

Chicks remained in the dark incubator after hatching for cca. 24 h without access to food or water. Afterwards, they were put in 25 cm × 25 cm boxes with one wall made of a mirror to reduce isolation stress. After 45 min of habituation a colored bead glued to the end of a 3 mm diameter rigid plastic tube was introduced to the chicks from above by the experimenter for 20 s. The tube was connected to a plastic syringe filled with tap water. When the chicks pecked at the bead, water droplets were administered through the bead as a reward. The presentation of the water reinforced bead was repeated two more times with 5-min intertrial intervals. For the fourth trial a bead with a different color was introduced, which had previously been dipped into methyl anthranilate (MeA), a bitter tasting substance, as negative reinforcement. All chicks that pecked on the MeA-covered bead showed a clear disgust response (beak opening, retreating, head shaking). Chicks that did not peck on the water beads at least two times out of the three trials, or failed to peck at the MeA bead, were excluded from the analysis. The color of the two types of stimuli alternated among individuals to avoid any effect of innate color preference (Zachar et al., 2008). Four hours later, the chicks were tested on dry beads of the same colors: MeA bead first, then the water bead with a 5-min intertrial interval. Number of pecks were recorded at every trial.

From day 2 onwards, the chicks were kept in their home cages heated by infrared light bulbs, in groups of 6 animals under a 12/12-h dark/light cycle with water and food available *ad libitum*.

#### Isolation Test (Day 2)

The chick was placed into a 42 cm × 34 cm × 44 cm cardboard box for five and a half minutes. The box then was covered with a Samsung Syncmaster 710v LCD monitor which also served as illumination. The behavior and the vocalization of the animal were recorded by a digital video camera with a wide angle lens through a small hole at one upper corner of the box. Through a 20 cm × 25 cm window on one side of the box another LCD monitor was visible. 150 s after the start of the session a 6 cm wide silhouette of a flying common buzzard (*Buteo buteo*) appeared on the overhead screen moving across for 4 s. After another 120 s a real size video recording of 5 age matched chicks was started to be played at the side monitor for 60 s. The experimental video recordings were analyzed by using Solomon coder (András Péter, http://solomoncoder.com). The recorded variables were: the latency of the first move and first distress call, the time spent moving, number and time of escape jumps and defecations. The precise time, number and amplitude of vocalizations were identified by a detailed analysis of the sound files extracted from videos (sampling frequency: 48,000 samples/s, quality: 16 bits).

For the sound analysis, we used the Seewave package (Sueur et al., 2008) in R (R Core Team, 2018). First, the spectrogram was generated (FFT length: 512, overlap: 50%) and all chicken calls were searched in the recordings. We characterized the calls by the amplitude of the strongest frequency based on the generated spectrogram, and the time position at that point. Only calls more intense than −45 dB were taken into account, where 0 dB corresponds to the amplitude of a 1 kHz reference signal (generated with maximum amplitude). The −45 dB threshold was chosen because the sound pressure of the noise generated by the movement of the chickens was less than this value. We managed to automatize the process using the R script. All automatic measurements were checked by visual inspection to exclude the potential errors of the automatic process. The −45 dB threshold served as a minimum reference level, and, in the following calculations, the intensity of calls were compared to this level resulting in sound pressure levels potentially ranging between 0 and 45 dB. The amplitude of vocalizations had been multiplied by the number of vocalizations in 10-s periods to obtain a variable characteristic to both the number and the loudness of the vocalizations in every 10 s of the experiment.

#### Social Preference Tests

##### Larger Versus Smaller Groups (Day 5)

**Figure 1:**
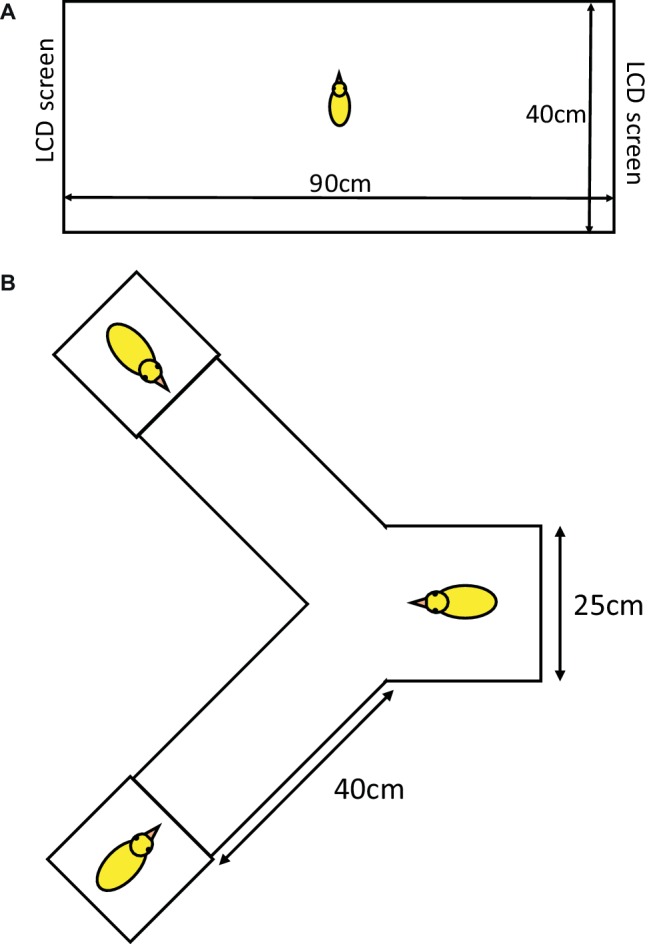
Experimental setup for the runway tests **(A)** and the Y-maze test **(B)**.

On post-hatch day 5, chicks were placed into a 90 cm × 40 cm runway apparatus (Figure 1A) facing one of the longer walls. Video recordings of three and eight chicks were played on Samsung Syncmaster 710v monitors at the opposite ends of the runway. The sides of the small group and large group video were alternating between subsequent individuals to control for any side preference. The runway was divided into three equal compartments (large group compartment, central compartment, and small group compartment). The test was recorded by an overhead camera and the videos were analyzed using the Solomon coder. Time spent in each part, as well as the first choice (touching one of the monitors), the latency to reach one end of the runway and the switches between the two stimulus chicks after the first choice were recorded. The test lasted for 4 min. Chicks that did not leave the central compartment during the test were excluded from the analysis.

##### Normal Versus Distorted Video Stimuli (Day 7)

On post-hatch day 7, chicks were placed into the same runway apparatus (Figure 1A) that had been used on day 5, facing one of the longer walls. Real size motion picture images of five chicks were played on monitors fixed at opposite ends of the runway. The two recordings were exactly the same except that, on one side, the facial characters (eyes and beak) were blurred on all chicks (using a digital video editing software). The sides on which the blurred and normal movies were played alternated between subsequent individuals to control for any side preference. For technical reasons, no video recordings were available for this test. The first choice of the chicks (touching one of the monitors) was recorded by the experimenter. The test lasted for 4 min. Chicks that did not leave the central compartment during the test were excluded from the analysis.

##### Choice Between Isolation-Reared and Group-Reared Individuals (Day 9)

On post-hatch day 9, the chicks were placed into a Y-maze (Figure 1B) to choose between two 9-day-old unfamiliar chicks. These were placed into 25 cm × 25 cm goal boxes at the end of the two arms of the maze. The boxes were separated from the maze by transparent glass walls. One of the stimulus chicks had been kept alone, isolated from the other conspecifics since hatching, meanwhile the other chick was kept in a group of six before the experiments. The experimental chick was placed at the starting box which lay 40 cm apart from each goal box. The test was recorded by an overhead camera and the videos were analyzed using the Solomon coder. Latency of first touch of the glass wall, the first choice, and the switches between the two stimulus chicks after the first choice were recorded. The test lasted for 4 min. Chicks that did not leave the central compartment during the test were excluded from the analysis.

##### Choice Between Familiar and Unfamiliar Individuals (Day 21)

On post-hatch day 21, the chicks were placed into a 45 cm × 160 cm runway facing one of the longer walls. In a goal box at one end of the runway, three familiar individuals (chicks that had been kept in the same home cage with the experimental chick from post-hatch day 2) were placed. In the goal box at the other end of the runway 3, unfamiliar individuals had been placed behind a wire mesh. The experimental chicks were allowed to spend 4 min in the runway. The target animals were swapped around between the two goal boxes in subsequent tests to control for any lateral preferences, and to keep the birds more alert. VPA-treated subject chicks were always tested with VPA-treated target (reference) chicks, while control subjects with control targets in both goal boxes; therefore, the only difference between the two sides was the factor of familiarity. After being used as target chicks, the animals were returned to their home cage for at least 90 min before they were used again as subjects for testing.

### Statistical Analysis

The sample size of each experimental group varied among the different experiments, since chicks that were passive in the preference tests or in the taste aversion learning test were omitted from the analysis. There was no significant difference between the two groups (VPA-treated and control) in the number of passive individuals in any of the tests, and there were no consistently passive individuals across the tests. The two experimental groups were compared using Student’s *t*-tests, when the variances were equal and Welch’s *t*-test when they were not. The effect of predatory stimulation on the distress call intensity in the open field test was assessed by repeated measures ANOVA with the treatment as independent factor. In the social preference tests, *χ*^2^ tests of homogeneity were used, for establishing whether the number of individuals choosing between the targets was different from random choice.

## Results

### General Observations

Success rate of hatching was 41/42 eggs in the control group, while the VPA-treated eggs hatched at a poorer rate of 23/32. Five out of the 23 VPA-treated chicks showed visible motor disorders (also noted by Nishigori et al., 2013), including serious muscle tone impairment: some of these chicks held their legs in an unnatural position, and were not able to move properly. In all impaired chicks, muscle weakness also appeared: some chicks were able to stand only for a few seconds, then they always sat down. All of the five chicks affected by motor deficits were excluded from the experiments. There was no apparent difference between the remaining VPA chicks and control chicks in their motor behavior; all birds showed intact righting reflex: they stood up within 2 s after being laid flat on their backs.

### Taste Aversion Learning and Positive Reinforcement (Day 1)

**Figure 2:**
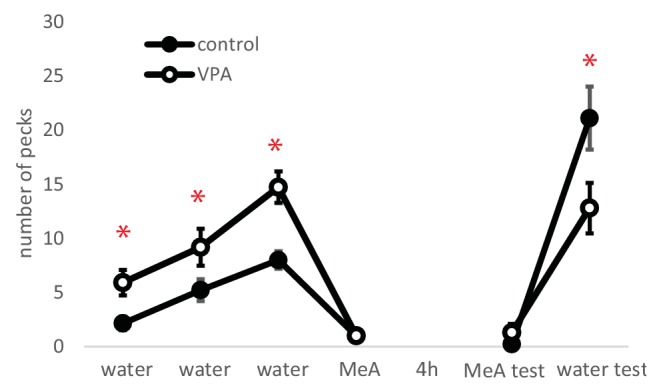
Number of pecks on target beads in taste aversion learning (mean ± s.e.m.). Asterisks denote significant differences between the VPA and control groups.

VPA-treated chicks pecked more on water-reinforced beads than control chicks (Student’s *t* > 2.59, d.f. = 26, *p* < 0.05, Figure 2) during the first three training trials. Both groups stopped pecking at the MeA-covered bead after tasting the bitter substance (Figure 2). Chicks that failed to peck in at least two out of the three positive training trials, or in the MeA trial, were excluded from further analysis. When tested 4 h later, both groups avoided the dry bead with the same color as the MeA-covered bead and readily pecked at the other dry bead with the color of the water-reinforced stimulus (Figure 2). However, control chicks tended to peck more frequently on the positively reinforced bead than did the VPA chicks (*t* = 2.25, d.f. = 17, *p* = 0.038, Figure 2). Both VPA and control chicks were able to discriminate between the positively and negatively reinforced stimuli after 4 h: every chick pecked more on the water-reinforced bead than on the MeA-reinforced one (VPA: *t* = 5.7, d.f. = 9, *p* < 0.001; control: *t* = 7.1, d.f. = 8, *p* < 0.001, Figure 2).

### Isolation Test (Day 2)

There was no difference between the VPA and control chicks in the motor activity (time spent moving: Student’s *t* < 0.46, d.f. = 36, *p* > 0.648) or in the latency of first move either after the start of the isolation (Student’s *t* = 0.86, d.f. = 36, *p* = 0.395) or after the onset of the predatory stimulus (Student’s *t* = 0.33, d.f. = 36, *p* = 0.74). Both groups showed an almost equal amount of escape behavior (jumping: Student’s *t* = 0.4, d.f. = 36, *p* = 0.675) and defecation (Student’s *t* = 1.71, d.f. = 36, *p* = 0.095) during the isolation, suggesting a similar distress level.

**Figure 3:**
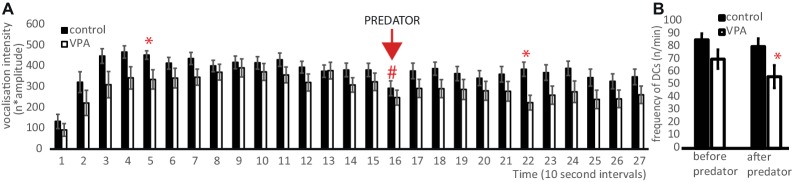
**(A)** Vocalization intensity during the isolation test in 10-s intervals (mean ± s.e.m.). Asterisks represent significant difference between VPA and control groups. Hash mark denotes significant difference compared to the previous 10-s intervals in both experimental groups. **(B)** Frequency of distress calls (DCs) during the isolation test (mean ± s.e.m.). Asterisks represent significant difference between VPA and control groups.

VPA chicks tended to emit fewer and/or less intense vocal calls than control chicks, however, these differences were significant only in two 10-s periods (T5, T22, Figure 3A) over the whole duration of the isolation experiment (Student’s *t* > 2.3, d.f. = 36, *p* < 0.05). The *p* here is statistically uncorrected for the large number of comparisons. Both VPA and control chicks reacted to the predator with a reduced vocalization in the subsequent 10 s (repeated measure ANOVA: *F* = 8.77, d.f. = 36, *p* = 0.005, Figure 3) and their reaction did not differ (*F* = 0.46, d.f. = 36; *p* = 0.832). However, if we selectively calculated the frequency of high-intensity (20 dB<) vocalization activity (representing distress calls) within the two stages of the experiment (before and after the presentation of predator), there was significant difference between the two groups in the latter stage (*t* = 1.72, d.f. = 35, *p* = 0.094; *t* = 2.20, d.f. = 35, *p* = 0.037, respectively, Figure 3B).

### Social Preference Tests

#### Larger Versus Smaller Groups (Day 5)

**Figure 4:**
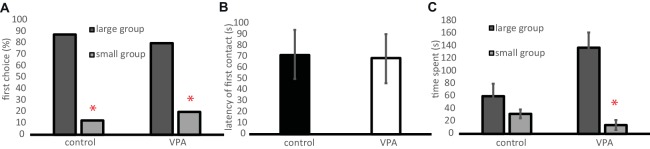
**(A)** Percentage of chicks approaching one end of the runway as their first choice between video recordings of a large group vs. a small group of chicks. Asterisks denote a choice significantly different from random choice. **(B)** Latency of the first choice (physical contact with the screen, mean ± s.e.m). **(C)** Time spent at each end of the runway (mean ± s.e.m). Asterisk denotes significant difference between the times spent in the proximity of the two screens.

The VPA treatment did not affect the preference toward a larger group of conspecifics over a smaller one as measured when the chicks were 5 days old. Approximately 80% of both VPA and control chicks chose the larger group as a first choice and the two groups did not differ in their choice (*χ*^2^ = 0.18, *n* = 18, *p* = 0.671, Figure 4A). The latency of the first choice also did not differ between the two groups (*t* = 0.78, d.f. = 16, *p* = 0.939, Figure 4B), suggesting similar levels of social motivation in the two groups. VPA chicks spent more time at the proximity of the larger group than of the smaller group (paired *t*-test: *t* = 4.76, d.f. = 11, *p* < 0.001, Figure 4C), whereas, in the controls, the difference between the time spent with the larger and smaller groups failed to reach statistical significance (paired *t*-test: *t* = 1.24, d.f. = 10, *p* = 0.243, Figure 4C). Notably, the latter is likely due to a tendency for control chicks to explore both ends of the runway more intensely through frequent switches of position between the target screens after their primary choice. Such difference in switching frequency becomes significant in later tests with older animals (see below).

#### Preference for Normal Over Distorted Social Stimuli

**Figure 5:**
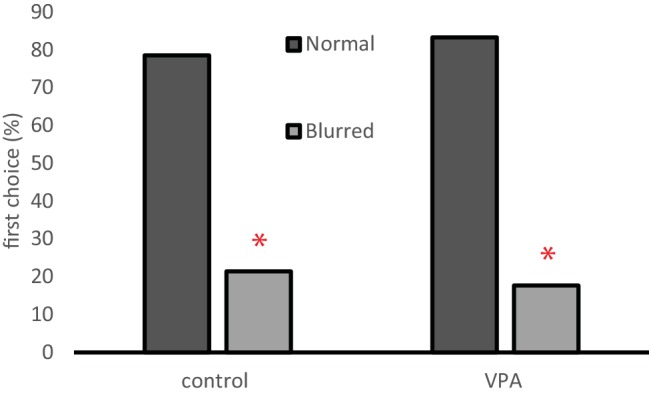
Percentage of chicks approaching one end of the runway as their first choice between video recordings of normal individuals vs. digitally distorted ones (head blurred). Asterisks denote a choice significantly different from random choice.

One-week-old chicks were tested whether they prefer video movies of normal conspecifics over the same movie without visible facial features (blurred head). Both groups were more inclined to choose the normal social stimulus (control: *χ*^2^ = 4.57, *n* = 14, *p* = 0.033; VPA: *χ*^2^ = 5.3, *n* = 12, *p* = 0.021, Figure 5).

#### Preference Toward Socialized Chicks Over Isolated Ones

**Figure 6:**
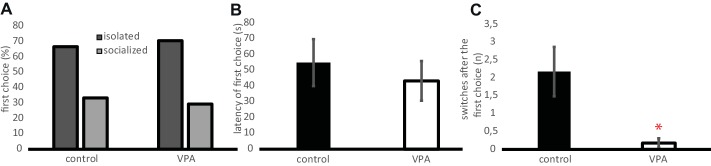
**(A)** Percentage of chicks approaching one end of the Y-maze as their first choice between a socially reared (socialized) chick and another one reared in isolation (isolated). The first choice of the VPA and control chicks did not differ significantly. **(B)** Latency of the first choice (physical contact with goal box, mean ± s.e.m.). **(C)** Number of positional switches performed by the experimental chicks between the two goal boxes after the first choice (mean ± s.e.m.). Asterisk denotes significant difference between VPA and control chicks.

To test whether VPA affects the detection of subtle details in the behavior of conspecifics at the age of 9 days, chicks were tested whether they prefer normally socialized conspecifics over those reared in isolation. Neither group showed significant preference toward any of the stimulus types as a first choice (control: *χ*^2^ = 1.64, *n* = 22, *p* = 0.201; VPA: *χ*^2^ = 2.88, *n* = 17, *p* = 0.09, Figure 6A). There was no difference between the two groups in the latency of the first choice either (*t* = 0.6, d.f. = 36, *p* = 0.554, Figure 6B). Both groups tended to spend more time at the proximity of the isolated chicks (VPA: 57.9 ± 9.9%, control: 55.4 ± 7.1%) than of the group-reared ones (VPA: 27.2 ± 9.7%, control: 31.1 ± 7.0%) but the difference was subsignificant. The two groups did not differ in their time spent at any of the stimulus chicks (*t* = 0.2, d.f. = 37, *p* = 0.841). However, chicks in the control group often abandoned their first choice and explored the other stimulus individual as well, while the VPA-treated chicks tended to stick to their first choice. The number of positional switches between the two stimulus individuals was significantly greater in the control group (Welch’s *t* = 2.67, d.f. = 22.45, *p* = 0.014, Figure 6C).

#### Recognition of and Preference for Familiar Individuals Over “Strangers”

**Figure 7:**
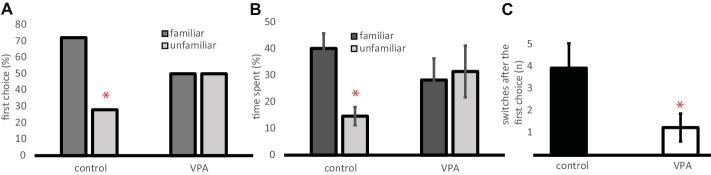
**(A)** Percentage of chicks approaching one end of the runway as their first choice between a familiar and an unfamiliar chick. Asterisk denotes a choice significantly different from random choice. **(B)** Time spent at each end of the runway (mean ± s.e.m.). Asterisk denotes significant difference between the two goal boxes. **(C)** Number of positional switches between the two goal boxes performed by the experimental chicks after their first choice (mean ± s.e.m.). Asterisk denotes significant difference between VPA and control chicks.

Three-week-old chicks preferred their sympatric, familiar conspecifics: they chose them primarily (*χ*^2^ = 4.84, *n* = 25, *p* = 0.028, Figure 7A) and spent more time in their proximity (*t* = 2.41, d.f. = 25, *p* = 0.024, Figure 7B). VPA-treated chicks failed to develop such a preference at the age of 3 weeks; they chose randomly between familiar and unfamiliar individuals (*χ*^2^ = 0.91, *n* = 12, *p* = 0.768, Figure 7A) and spent an equal amount of time in their proximity (*t* = 0.31, d.f. = 11, *p* = 0.76, Figure 7B). Similar to the previous tests, control chicks tended to explore the goal boxes at both ends of the runway: they switched sides 3–4 times more often after the first choice than did VPA-treated chicks (*t* = 2.1, d.f. = 28, *p* = 0.046, Figure 7C).

## Discussion

Newly hatched domestic chicks have often been used as models in studies of behavioral neuroscience (Bolhuis and Honey, 1998; Rose, 2000; Zachar et al., 2008), because, despite their young age, they are capable of displaying complex behaviors, while, at the same time, an extensive previous experience does not interfere with their behavior (Rose, 2000). Chicks prefer the close proximity of conspecifics. One widely used behavioral paradigm is filial imprinting (Horn, 1998; Matsushima et al., 2003), which is not entirely devoid of social context, since it is the trigger of social bond forming between parent and offspring. Chicks also react to social isolation by displaying behaviors aimed at reuniting with conspecifics (Gallup and Suarez, 1980), and they prefer larger groups of siblings over smaller ones (Zachar et al., 2016). The drive to reinstatement can be evaluated by measurement of distress vocalization (Takeuchi et al., 1996; Yazaki et al., 1999). Such innate gregariousness likely relies on the social brain network, since recognition of and exposure to just one same-age conspecific partner activates brain regions of the social brain network in naïve domestic chicks (Mayer et al., 2017). This suggests that, even if lacking time to form social bonds, affiliation to siblings is likely processed similarly to other social behaviors.

The present findings seem to confirm previous reports concerning an adverse effect of VPA on embryonic development, including a tendency for aborted or delayed hatching and, occasionally, for locomotor disorders. However, those VPA chicks that have overcome these initial deficits will perform normally in locomotor tests and remain in good physical condition. Judged by visual observation (not quantified, off-record) over the entire period of experiments, VPA chicks tend to display normal social behaviors not overtly dissimilar from control peers (aggregation, synchronized activities such as joint sleeping and waking cycles, joint pecking at objects). Also, their distress level appears to be similar to that of controls, apart from minor differences at selected periods of monitoring. In agreement with the results on filial imprinting in previous reports (Nishigori et al., 2013; Sgadò et al., 2018), appetitive (water reward) and taste aversion (MeA) learning, as well as discriminative learning (recognizing the color bead) of chicks are unaffected by embryonic VPA treatment. Whereas cognition remains unimpaired, the observed elevation of pecking frequency during positive water reinforcement training in VPA chicks may be due to an increased tendency of stereotypic pecking. The latter is known to be related to dopaminergic overstimulation, also in birds (Kabai et al., 1999; Zsedényi et al., 2014). Repetitive and stereotypic behaviors are among the standard symptoms of human ASD (Bangerter et al., 2017). On the other hand, this does not explain the observed elevation of pecking of control birds during the test phase. Another viable hypothesis to explain the increased pecking on water reinforcement is in line with the observation of excessive water consumption in autistic humans (Mills and Wingy, 2015).

The existing reports on the early behavioral effects of embryonic VPA treatment of domestic chicks in relation to ASD (Nishigori et al., 2013; Sgadò et al., 2018; Lorenzi et al., 2019) seem to agree upon the point that VPA does not impair filial imprinting. In line with this is our observation that another form of early adaptive learning that is devoid of social context (passive avoidance learning) is not attenuated by VPA either (present study). However, at variance with the reports by Sgadò et al. (2018) and Lorenzi et al. (2019), we did not find significant differences between VPA-treated and control birds in other behavioral tasks relevant to early social preferences: distress calls evoked by isolation (suspended by predator sighting), and approach preference for larger over smaller group of conspecifics (with natural or blurred facial features), at least, in the period between 1 and 9 days of age.

It has to be noted that, in the study by Nishigori et al. (2013), vocalization of the VPA exposed birds was found to be reduced. In our case, such an overall reduction was suggestive as a trend but not significant, except for high-intensity (distress) calls in selected periods of observation. However, the response to predator sighting (abrupt silencing of distress calls) was uniformly present both in the experimental and in the control group, indicating that the predisposition of chicks to avoid danger from overhead attacks was unaffected by VPA exposure.

The apparent contradiction between the report of Sgadò et al. (2018) and our current findings concerning susceptibility of innate preferences (predispositions) to embryonic VPA challenge can likely be ascribed to meaningful differences in the experimental conditions. In the cited study, social predisposition of naïve, dark-reared chicks was assessed by a choice between two stimuli of “face configuration” (a stuffed hen or a scrambled version of it), on post-hatch day 2, whereas imprinting to similar objects was carried out no later than day 3. In our study, the earliest measure of innate behavior was distress vocalization evoked by social isolation on post-hatch day 2, followed by an approach preference for larger groups of conspecifics on day 5. Thus, in our case, the modality and salience of the stimuli triggering the behavior clearly differed from those applied in the study by Sgadò et al. (2018).

While the current findings do not suggest an impairment of innate preferences by VPA exposure in the categories studied (however, they do not preclude the existence of such effect in other categories), our results point to an important novel aspect of VPA-dependent alterations of sociability, developing during the first 3 weeks of life. The first subtle sign of disturbance emerged at day 9, when VPA-exposed chicks showed reduced exploration of conspecifics (while they still did not differ significantly from controls in their choice between socially reared and isolation-reared partners). The intensity of social exploration (number of times the birds switch proximity position between group-reared and isolation-reared partners) proved to be a useful and important parameter, potentially indicating behavioral plasticity and flexibility in control chicks, as opposed to rigidity and perseverance in VPA-exposed chicks. Notably, 3-week-old chicks showed a similar difference between the numbers of positional switches in the familiarity tests (again, control birds outperforming VPA birds). Presumably, the reduced number of side switches in VPA chicks cannot be ascribed to impaired locomotor activity, since it has been found to be unaffected in running wheel test (Nishigori et al., 2013) or by measuring open field activity (in the present study). Still, it cannot be excluded that the observed deficit affected general, rather than just socially driven, exploration.

The results of the present study answer an important, hitherto unadressed, question raised by the work of Nishigori et al. (2013) by suggesting that the observed deficits in group-forming and group-cohesion were due to attenuated motivation for exploring social stimuli, rather than to impaired stimulus recognition. It is expected that, without proper exploration, the individual recognition required for the complex social life of adult domestic chicks would fail to develop by the third week.

At 3 weeks of age, by which time the control birds have acquired the capability of distinguishing familiar sympatric birds from non-familiar partners (Koshiba et al., 2013), the VPA-exposed chicks perform markedly poorer in this task. It has to be noted that, in our study, we did not attempt to determine the precise nature of the “familiarity stimulus.” According to the study by Koshiba et al. (2013), familiar recognition in domestic chicks of 15 days of age is based primarily on acoustic (rather than visual or olfactory) cues. According to the above-cited authors, it is unknown if chicks are capable of recognizing familiar peer calls based on individual chick recognition. Nevertheless, visual cues underlying the recognition of familiar conspecifics of chicks have been reported to appear well before that period (Vallortigara, 1992; Vallortigara and Andrew, 1994), and, on the whole, conspecific recognition can likely be ascribed to a complex multimodal input from primary and reinforcing stimuli (Bolhuis, 1991), and a reciprocal communication between subject and target.

In summary, VPA administration on the 14th day of incubation *in ovo* impaired certain acquired (primarily exploratory) social behaviors and social memory of young post-hatch chicks, but it failed to cause robust defects in their hardwired predispositions. The most prominent findings included an attenuation of social exploration, followed by a remarkable deficit in the recognition of familiar conspecifics, unfolding at the end of the third week after hatching. These novel results underline the importance of further investigation into the differences in early social exploration, potentially contributing to the early diagnosis of ASD.

## Data Availability

The datasets generated for this study are available on request to the corresponding author.

## Ethics Statement

All animals were kept and treated according to the regulations of the ethical committee of the Semmelweis University, and all experiments were approved by the Ethical Committee on Animal Experimentation, and permitted by the Food Chain Safety and Animal Health Directorate of the Government Office for Pest County (Permit Number: XIV-I-001/2269-4/2012). Procedures were in harmony with the EU Council directives on laboratory animals (86/609/EEC).

## Author Contributions

GZ, AT, and AC designed the experiments. AT was mostly involved in the technical execution of behavioral tests. GZ, AT, and SZ analyzed the video recordings. AT, LG, and ÁÁ carried out the embryonic manipulations. The manuscript was written and finalized by GZ and AC.

### Conflict of Interest Statement

The authors declare that the research was conducted in the absence of any commercial or financial relationships that could be construed as a potential conflict of interest.
